# Metastasis-associated protein 1 (MTA1) is transferred by exosomes and contributes to the regulation of hypoxia and estrogen signaling in breast cancer cells

**DOI:** 10.1186/s12964-019-0325-7

**Published:** 2019-02-19

**Authors:** Bethany N. Hannafon, Amy L. Gin, Yi-Fan Xu, Matthew Bruns, Cameron L. Calloway, Wei-Qun Ding

**Affiliations:** 1 Department of Pathology, University of Oklahoma Health Sciences Center, 975 NE 10th Street, BRC 411A, Oklahoma City, OK 73104 USA; 2 Peggy and Charles Stephenson Cancer Center, Oklahoma City, OK USA

**Keywords:** Exosomes, MTA1, Breast cancer

## Abstract

**Background:**

Exosomes are small membrane-bound vesicles that contribute to tumor progression and metastasis by mediating cell-to-cell communication and modifying the tumor microenvironment at both local and distant sites. However, little is known about the predominant factors in exosomes that contribute to breast cancer (BC) progression. MTA1 is a transcriptional co-regulator that can act as both a co-activator and co-repressor to regulate pathways that contribute to cancer development. MTA1 is also one of the most up-regulated proteins in cancer, whose expression correlates with cancer progression, poor prognosis and increased metastatic potential.

**Methods:**

We identified MTA1 in BC exosomes by antibody array and confirmed expression of exosome-MTA1 across five breast cancer cells lines. Ectopic expression of tdTomato-tagged MTA1 and exosome transfer were examined by fluorescent microscopy. CRISPR/Cas9 genetic engineering was implemented to knockout MTA1 in MCF7 and MDA-MB-231 breast cancer cells. Reporter assays were used to monitor hypoxia and estrogen receptor signaling regulation by exosome-MTA1 transfer.

**Results:**

Ectopic overexpression of tdTomato-MTA1 in BC cell lines demonstrated exosome transfer of MTA1 to BC and vascular endothelial cells. MTA1 knockout in BC cells reduced cell proliferation and attenuated the hypoxic response in these cells, presumably through its co-repressor function, which could be rescued by the addition of exosomes containing MTA1. On the other hand, consistent with its co-activator function, estrogen receptor signaling was enhanced in MTA1 knockout cells and could be reversed by addition of MTA1-exosomes. Importantly, MTA1 knockout sensitized hormone receptor negative cells to 4-hydroxy tamoxifen treatment, which could be reversed by the addition of MTA1-exosomes.

**Conclusions:**

This is the first report showing that BC exosomes contain MTA1 and can transfer it to other cells resulting in changes to hypoxia and estrogen receptor signaling in the tumor microenvironment. These results, collectively, provide evidence suggesting that exosome-mediated transfer of MTA1 contributes to BC progression by modifying cellular responses to important signaling pathways and that exosome-MTA1 may be developed as a biomarker and therapeutic target for BC.

**Electronic supplementary material:**

The online version of this article (10.1186/s12964-019-0325-7) contains supplementary material, which is available to authorized users.

## Background

Exosomes are small (50-100 nm) secretory vesicles that mediate cell-to-cell communication in the tumor microenvironment by encapsulating and transferring cancer-promoting factors to surrounding cells or to distant sites through the circulation. Studies have demonstrated that breast cancer (BC) cells subjected to hypoxic conditions secrete exosomes in significantly greater numbers [1], and that exosomes shed from hypoxic BC cells promote focal adhesion formation, invasion, and metastasis [2], thus implicating that cancer exosomes are mediators of tumor metastasis. However, the predominant factors in exosomes that promote these processes are currently not fully understood.

The metastasis-associated proteins are a family of co-regulators comprised of MTA1, MTA2, and MTA3. MTA members are critical components of the nuclear remodeling and deacetylation (NuRD) complex and are primarily involved in regulating target gene expression through deacetylation of histones in chromatin [3]. As a major co-regulatory molecule MTA1 can quickly respond to physiological or developmental cues by altering gene expression in opposing directions. MTA1 is over-expressed in many cancers, including BC, and correlates with tumor metastasis and progression in human BC [4–8] and transgenic mouse models [9]. It is believed that the effects of MTA1 are due to its regulation of various cancer-promoting processes, including estrogen receptor signaling [10], canonical Wnt1/β-catenin signaling [11], hypoxia-inducible factor-1α (HIF-1α) stabilization [12, 13], and regulation of EMT via repression of E-cadherin and other adhesion molecules [14, 15]. MTA1 is also involved in the DNA-damage response, and rapidly accumulates at sites of DNA damage [16]. Other studies have shown that MTA1 may have a variety of roles independent of the NuRD complex [8]. Despite these documented roles, specifically how MTA1 induces metastasis in cancer is unclear.

Using a cancer biomarker antibody array, we identified MTA1 protein in BC exosomes that may regulate hypoxia and estrogen signaling and contribute to BC progression. We characterized the exosome-mediated transfer and mechanistic role of exosome MTA1, by overexpression using fluorescent-tagged MTA1 protein and genetic loss of MTA1 using CRISPR/Cas9 genetic engineering methods. The role of exosome MTA1 on estrogen and hypoxia signaling mechanisms via exosome-mediated transfer were also investigated. This work indicates that exosome transfer of MTA1 protein may contribute to BC development and progression.

## Methods

### Cell culture

The normal human breast cell line MCF10A, and breast cancer cell lines MCF7, MDA-MB-231, ZR-75-1, BT20, and SK-BR-3 were obtained from the American Type Culture Collection (Manassas, VA). The MCF7, MDA-MB-231, ZR-75-1 and BT-20 cells were grown in DMEM medium supplemented with 10% fetal bovine serum (FBS), 100 IU/mL penicillin and 100 μg/mL streptomycin (Corning/Mediatech, Inc. Manassas, VA). The MCF10A cells were cultured in DMEM/F12 supplemented with 5% horse serum, 20 ng/ml epithelial growth factor, 0.5 mg/ml hydrocortisone, 100 ng/ml cholera toxin, 10 μg/ml insulin, 100 IU/ml penicillin, and 100 μg/ml streptomycin. SK-BR-3 cells were cultivated in McCoy’s 5A medium supplemented with 10% FBS, 100 IU/ml penicillin and 100 μg/ml streptomycin. Exosome-depleted FBS and horse serum were prepared by pelleting the serum exosomes by ultracentrifugation at 100,000×g for 2 h at 4 °C, and the resulting supernatant was filtered through a 0.2-μm pore filter. Cells were routinely maintained in a humidified chamber at 37 °C and 5% CO_2_.

### Exosome isolation

Exosomes were isolated by sequential centrifugation, filtration and ultracentrifugation as we have previously reported [17]. Exosome concentration was determined using the Pierce BCA protein assay (Thermo Fisher Scientific).

### Cancer biomarker antibody Array

Exosome lysates from MCF7 cells were prepared, biotinylated, and incubated with the Cancer Biomarker Antibody Array (Cat. No. SCB200, Full Moon Biosciences) according to the manufacturer’s instructions. The proteins were detected using Cy3-streptavidin (Thermo Fisher Scientific). Fluorescent intensity was measured at 532 nm on an Agilent Sure Scanner (Agilent Technologies). Fluorescent signal intensity was normalized to the mean of the positive control signal intensities according to the following equation: normalized signal intensity = (mean signal intensity for protein X) x (mean signal intensity of positive control on reference array)/mean signal intensity of positive control on array Y). Enriched gene ontology terms among proteins identified on antibody array were determined by the C5 GO gene set collection from the Molecular Signature Database v6.1 (MSigDB) [18] using the Gene Set Enrichment Analysis Software program [19].

### Stable expression of CD63 and MTA1

The generation of MCF7 and MDA-MB-231 cells overexpressing GFP-tagged CD63 was previously reported [17]. The lentiviral vector pLV-Neo-CMV > ORF_1431bp:3xGGGGS:hMTA1[NM_004689.3] (lenti-CMV-tdTom-MTA1) used to overexpress tdTomato-tagged full length MTA1 isoform was constructed by VectorBuilder (Cyagen Biosciences). The vector ID is VB151117–10065, which can be used to retrieve detailed information about the vector on www.vectorbuilder.com.

### Single guide RNA design and CRISPR/Cas9 knockout

The online guide design tool from DNA 2.0 gRNA (now known as ATUM, Newark, CA) was used to identify sgRNAs. The DNA sequence corresponding to the annotated stem loop miRNA (www.miRBase.org) was used as an input sequence. The highest scoring guides and/or those closest to the mature miRNA sequence were selected. Complementary oligos containing the sgRNA sequence and *BsmBI* overhangs were synthesized (Integrated DNA Technologies), annealed, digested with *BsmBI* and ligated into the lentiCRISPR v2, a gift from Feng Zhang (Addgene, # 52961) [20]. MTA1-sgRNA-1: 5′- CTCCAAGGCCATCTCGGCGC-3′; MTA1-sgRNA-3: 5′- CAGCTGCGGCGCTCATGTGC-3 and MTA1-sgRNA-5: 5’-CTCTGTGGGCACCTTCGCAC-3′. MCF7 and MDA-MB-231 cells were infected with lentivirus in the presence of 8 μg/ml polybrene (Sigma-Aldrich). Approximately 48 h post-infection cells were selected by treating with 1 μg/ml puromycin (InvivoGen, San Diego, CA) for 3 days.

### Lentiviral transduction

Lentiviral particles were produced similarly as before [17] using the 3rd generation packaging plasmids pMD2.G (Addgene plasmid #12259); pMDL/ RRE g/p (Addgene plasmid #12251) and pRSV-Rev (Addgene plasmid #12253) were a gift from Didier Trono. The packaging plasmids were co-transfected with the lentiviral expression vector into human embryonic kidney 293 T cells using the polyethyleneimine (Polysciences Inc.) transfection method to produce replication deficient lentivirus. After 48 and 72 h of transfection, supernatants were pooled, filtered through a 0.45-μm membrane and concentrated by ultracentrifugation at 100,000 x g. MCF7 cells were infected with lentivirus in the presence of 8 μg/ml polybrene (Sigma-Aldrich). Approximately 48 h post-infection cells were selected by treating with 400 μg/ml G418 (InvivoGen, San Diego, CA) for 7 days.

### Genomic PCR, T7 endonuclease assay, and sanger sequencing

Genomic DNA was extracted from wildtype and Cas9/sgRNA transduced and puromycin selected MCF7 cells using the Pure Link Genomic DNA Mini-kit (Invitrogen) according to the manufacturer’s protocol. Primers were designed to amplify a ~ 800 bp fragment surrounding the sgRNA cleavage site. MTA1 genomic primers: forward 5′- CTTGGCCGACACTGTGGT-3′ and reverse 5′- GACAGGAAGGACTATGGCGG-3′. The genomic loci of interest were amplified by PCR using Phusion High-Fidelity DNA Polymerase (Thermo-Scientific). The PCR amplicons were column purified using the MicroElute DNA cleanup Kit (Omega Bio-Tek). To assess the gene editing efficiency, the T7 Endonuclease assay was used. Briefly, 200 ng of purified PCR product was diluted in 1X NEB Buffer 2 (New England Biolabs) and reannealed using the following conditions: denaturation at 95 °C for 5 min, re-annealing by ramping down the temperature to 85 °C at a rate of 2 °C per second, then from 85 °C to 25 °C at a rate of 0.1 °C per second, and a final hold at 4 °C. Ten units of T7 Endonuclease I (T7EI) (New England Biolabs) enzyme was added to the annealed PCR products and incubated at 37 °C for 15 min. The reaction was inhibited by adding 1.5 μl of 0.25 M EDTA. The T7EI digestion products were visualized by running on an Agilent Bioanalyzer DNA 1000 Chip (Agilent Technologies). Successful editing was determined by the presence of T7EI cleaved products in the Cas9/sgRNA transduced cells compared to wildtype cells. Single cell clones of each transduced cell line were expanded and sequenced for mutation pattern determination. The PCR amplicons of each clone were cloned into the pCR™4-TOPO® TA vector (Thermo-Fisher). Random colonies were selected and sent for Sanger sequencing using the T7 primer (5’-TAATACGACTCACTATAGGG-3′).

### Western blot

Total cellular and exosome protein was prepared by re-suspending exosomes in RIPA buffer (50 mM Tris–HCl pH 7.4, 150 mM NaCl, 0.5% sodium deoxycholate, 1% NP-40, and 0.1% sodium dodecyl sulfate) containing 1X protease inhibitor cocktail (Protease Inhibitor Mini Tablets, Pierce). About 30–50 μg of protein was separated by SDS-PAGE (10%), transferred to a PVDF membrane and blotted with antibodies against MTA1, N-terminal (AB06723PU-N, OriGene), internal epitope (sc-17773, Santa Cruz Biotechnology), C-terminal epitope (5647, Cell Signaling Technology), GAPDH (20035, ProMab Biotechnologies, Richmond, CA), RFP (600–401-379, Rockland, Limerick, PA), and CD63 (sc-5275, Santa Cruz Biotechnology, Santa Cruz, CA).

### Luciferase reporter assays

The estrogen response element luciferase reporter (3X ERE TATA luc) containing three copies of vitellogenin Estrogen Response Element was a gift from Donald McDonnell (Addgene plasmid # 11354) [21]. The pGL3-HRE-luciferase reporter construct containing the hypoxia response elements (HRE) of the VEGF gene promoter was kindly provided by Dr. Konstantin Salnikow (Radiation Oncology Branch, NCI, Frederick, MD) [22]. MCF-7 and MDA-MB-231 cells were seeded into a 6-well plate and reached 70–80% confluence 24 h after plating. For estrogen signaling assay cells were plated in phenol-red free Improved Minimal Essential Media (IMEM) (Corning) supplemented with 5% charcoal-stripped fetal bovine serum (GeneTex). The cells were then transfected with 2.5 μg 3X ERE TATA luc or pGL3-HRE-luciferase along with the 1 μg renilla luciferase expression plasmid (pRLTK) as a transfection control using the Lipofectamine 3000 transfection reagent (Thermo Fisher Scientific) (16). The next day, cells were lifted and re-plated at a density of 10,000 cells per well in triplicate in white 96-well plate with or without exosomes. The following day the cells were treated with 150–300 μM cobalt chloride hexahydrate dissolved in PBS or 5–10 nM 17β-estradiol dissolved in ethanol (Sigma Aldrich) as appropriate. Approximately 16–24 h after treatment cells were lysed and assayed for luciferase activity using the Dual Luciferase Assay kit (Promega). Luminescence was detected on a Perkin Elmer Envision Multilabel reader. Data normalization was conducted by dividing firefly luciferase by renilla luciferase luminescence.

### RNA extraction and real-time PCR

Total RNA from cultured cells was prepared using the TRIzol Reagent (Invitrogen/Thermo-Fisher) followed by a column clean-up using the PureLink RNA Mini Kit according to the manufacturer’s protocol for whole transcriptome isolation (Invitrogen). RNA concentration was quantitated using the NanoDrop ND-100 Spectrophotometer (NanoDrop Technologies). Gene expression was measured by generating cDNA from 200 ng of total RNA using the iScript cDNA Synthesis kit (Bio-Rad, Hercules, CA). The synthesized cDNA was diluted in 2X iTaq Universal SYBR Green Supermix (Bio-Rad, Hercules, CA) and combined with 10 μM of each forward and reverse primer. Specific primer sequences used are as follows: IGFBP4, forward 5′- AGCCCTCTGACAAGGACGAG-3′ and reverse 5′- TCCGGTCTCGAATTTTGGCG-3′; SLC2A1, forward 5’-CTGGCATCAACGCTGTCTTC-3′ and reverse 5′- GTTGACGATACCGGAGCCAA-3′; and the normalization control 36B4, forward 5′-ATCAACGGGTACAAACGAGTCCTG-3′ and reverse 5′- AAGGCAGATGGATCAGCCAAGAAG-3′. PCR reactions were run on the Bio-Rad CFX 96 Real-Time PCR (Bio-Rad, Hercules, CA) instrument under the following conditions: hold at 95 °C for 10 min, then 40 cycles of 95 °C for 15 s and 60 °C for 1 min. Relative gene expression was assessed using the differences in normalized Ct (ΔΔCt method) after normalization to 36B4.

### MTS cell proliferation assay

Cells were seeded onto 96-well plate at a density of 12,000 cells/well in quadruplicate. The cells were cultured at 37 °C with 5% CO^2^ for 4 days. For each well, the attached cells were incubated in 100 μL growth medium supplemented with 20 μL CellTiter 96® AQueous One Solution (Promega, Madison, WI, USA) and incubated for 1 h. The absorbance value at 495 nm was recorded using a spectrometer.

### Fluorescent microscopic imaging

Cells co-expressing tdTom-MTA1 and CD63-GFP were grown on a glass coverslip to 50% confluence. For co-culturing exosome uptake imaging 1 × 10^4^ cells were plated in the upper chamber of a trans-well insert (0.4 μm pore size) and 1 × 10^5^ ZR-75-1 or EA.hy926 cells were plated on glass coverslips in the bottom chamber of a 24-well plate and cultured for 4 days. Cells were fixed with 4% paraformaldehyde for 15 min. Coverslips with fixed cells were mounted on glass slides using ProLong Gold Antifade with DAPI (Thermo Fisher Scientific). Confocal images were collected on a Leica SP8 Confocal White Light Laser system microscope using a 40X objective. Fluorescent images were collected on a Nikon TE2000-E microscope using the 40X objective. For co-localization analysis, four regions of interest (ROI) were drawn over areas of tdTomato and GFP signal overlap. The average co-localization within the ROIs was calculated by measuring the pixel intensity-correlation by Pearson coefficient using the coloc2 plugin for Fiji/ImageJ software.

## Results

### Breast cancer exosomes containing MTA1 promote hypoxic response

**Fig. 1:**
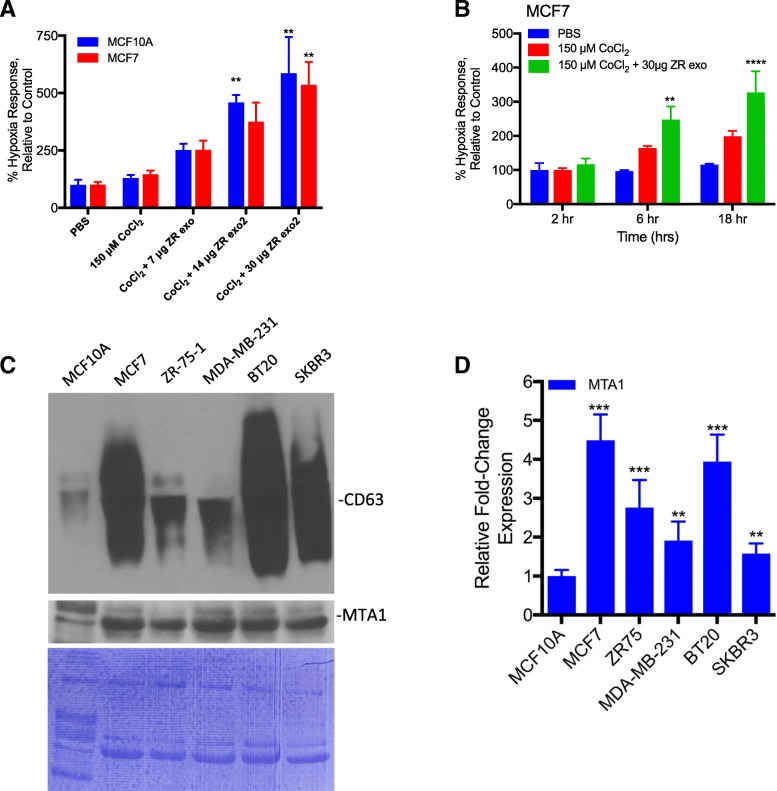
Exosomes from breast cancer cells promote hypoxia signaling and contain MTA1. **a**. Hypoxia response element (HRE) luciferase reporter assay. MCF10A and MCF7 cells were transfected with the HRE reporter plasmid and treated with increasing concentrations of exosomes from ZR-75-1 breast cancer cells. Hypoxic response was induced using cobalt chloride and cells were assayed for luciferase activity after 18 h of treatment *n* = 3, **p < 0.01 one-way ANOVA. **b**. MCF7 cells were transfected with the HRE reporter plasmid and treated with cobalt chloride and ZR-75-1 exosomes. Cells were assayed for luciferase activity at 2, 6 and 18 h n = 3, **p < 0.01, ****p < 0.0001, Two-way ANOVA. **c**. Western blot analysis of CD63 and MTA1 expression in exosomes isolated from normal breast cells (MCF10A) and breast cancer cell lines (MCF7, ZR-75-1, MDA-MB-231, BT20, SK-BR-3). Coomassie staining is included as an equal loading control. **d**. Real-time PCR analysis of fold-change in expression of cellular MTA1 mRNA across breast cancer cell lines relative to normal breast cells (MCF10A), *n* = 3, **p < 0.01, ***p < 0.001, Student’s t test

As exosomes have been shown to promote cancer progression and metastasis, we examined the role of exosome transfer on activation of hypoxia signaling. A hypoxic state was induced with cobalt chloride in MCF10A and MCF7 cells and the hypoxic response was monitored using a hypoxic response element-driven luciferase reporter. With the addition of exosomes from the breast cancer cell line ZR-75-1, the hypoxic response was increased in both a dose- and time-dependent manner (Fig. 1a, b). To understand what specific cancer-related proteins were present in breast cancer exosomes that might promote this observed effect, exosomes were isolated from the conditioned media of the normal breast cell line MCF10A and the estrogen receptor positive breast cancer line MCF7. The isolated exosomes were verified by western blot, electron microscope, and nanoparticle analysis as we recently described [17, 23]. Exosome lysates were prepared and probed with a cancer biomarker array containing antibodies against 247 established cancer markers. Out of the 247 antibodies on the array, 77 resulted in a positive signal among both samples, 7 proteins were specifically present in MCF10A exosomes, 56 were specifically present in MCF7 exosomes, and 14 were detected in both samples (Additional file 1). After data normalization, of the 77 proteins detected 64 were significantly (*p* < 0.05) differentially expressed in MCF7 exosomes relative to MCF10A exosomes with a fold-change of 1.5 or greater. As expected, the proteins significantly over-expressed in MCF7 exosomes include cytokines, growth factors, proteases, cytokine and growth factor receptors, cell adhesion molecules, extracellular matrix and cytoskeletal proteins (Table 1). To determine enriched gene ontology (GO) terms among the detected MCF7 exosome proteins we performed Gene Set Enrichment Analysis. As shown in Table 2, the most overrepresented GO terms included “extracellular space”, followed by “receptor binding”, and “response to oxygen containing compound”. Several of the proteins detected, however, were ascribed unexpected molecular functions including DNA binding proteins, transcription factors, and DNA repair proteins.

**Table 1:** Breast cancer exosome proteins identified by cancer biomarker antibody array. Proteins identified in either group with a fold-change ≥1.5 (MCF7 vs. MCF10A), *p* < 0.05 (Student’s T test) are listed and grouped by molecular function

Protein Name	Swiss Prot	Fold Change	*P*-value	Protein Name	Swiss Prot	Fold Change	P-value
*Signaling molecules (cytokines, growth factors, and peptide hormones)*				*Cytoskeletal proteins*			
Myostatin	O14793	21,111.91	2.29E-10	Tubulin gamma	P23258	754.10	1.93E-13
FSH	P01225	15,362.78	4.48E-11	Tubulin beta	Q13509	450.98	6.32E-11
IL-15	P40933	8289.69	9.00E-12	Beta actin		2.77	2.80E-09
IGF-II	P01344	1935.77	4.89E-12	Tubulin alpha	Q71U36	1.89	2.78E-02
VEGFB	P49765	1535.45	1.51E-10	*Protease inhibitor*			
IL-12	P29459	1072.56	1.28E-09	ACT	P01011	1573.14	2.07E-11
MIP-3β/CCL19	Q99731	838.61	1.36E-12	TIMP-1	P01033	1296.86	2.69E-10
TGFβ2	P61812	691.68	7.88E-15	PAI-1/SERPINE1	P05121	0.00007	1.50E-08
PDGFB	P01127	365.81	8.82E-13	*DNA Binding Protein*			
Mammaglobin B	O75556	285.27	7.06E-04	MLL/KMT2A	Q03164	402.38	6.22E-17
Osteopontin	P10451	262.26	7.16E-04	MSH2	P43246	175.50	1.15E-12
IL-17E	Q9H293	226.68	1.56E-12	MTA1	Q13330	168.22	1.51E-16
IGF-I	P05019	174.44	4.99E-02	*Cell Adhesion Molecules*			
IL-17F	Q96PD4	171.53	1.00E-11	CEA	P06731	4423.56	1.96E-11
TNF-alpha	P01375	107.92	5.67E-04	VCAM-1	P19320	573.18	4.07E-11
TGF alpha	P01135	81.47	4.95E-02	ALCAM	Q13740	344.25	3.76E-14
IL-13	P35225	0.0004	1.02E-02	ITGB2/CD18	P05107	0.12	1.31E-03
NGF	P01138	0.08	6.23E-13	TSP1	P07996	0.03	1.05E-15
Resistin	Q9HD89	0.05	8.90E-13	*Transfer/Carrier Protein*			
RANTES/CCL5	P13501	0.0002	3.28E-10	ALB	P02768	165.05	3.77E-12
EGF	P01133	0.0002	5.66E-04	AFP	P02771	14,293.40	3.98E-11
*Hydrolase/Protease*				*Extracellular Matrix Protein*			
PSA	P07288	7771.26	5.22E-15	COL4A1	P02462	2326.71	2.49E-06
MMP-10	P09238	7395.53	1.10E-10	Laminin		387.50	1.24E-13
MMP-2	P08253	4044.93	2.41E-06	COL3A1	P02461	320.58	3.22E-09
MMP-19	Q99542	298.36	3.60E-11	ITGA5	P08648	131.46	9.71E-12
MMP-7	P09237	186.87	1.07E-02	Fibronectin	P02751	0.26	8.82E-13
MMP-11	P24347	42.32	1.10E-10	*Oxidoreductase*			
*Cytokine receptor*				MPO	P05164	419.24	3.16E-10
TGFBR3	Q03167	902.09	3.53E-09	Defense/Immunity Protein			
TGFBR2	P37173	295.19	1.09E-08	B2M	P61769	0.060	8.91E-13
IL2RA	P01589	0.01	6.00E-10	IgA		0.067	8.99E-11
*Growth Factor Receptor*				*Storage Protein*			
CD40LR	P25942	519.22	1.08E-12	Ferritin	P02794	1414.57	3.53E-13
Tek/Tie2	Q02763	402.05	1.21E-10	*Transcription Factor*			
TYRO3	Q06418	245.33	5.91E-04	THR alpha	P10827	1164.74	1.75E-14
NTRK1/TrkA	P04629	236.47	6.48E-14	*Transferase/Kinase*			
PDGFR alpha	P16234	204.86	5.11E-11	Tyk2	P29597	15,893.91	8.85E-16
				*DNA Repair Protein*			
				MUM1	Q2TAK8	632.43	5.45E-04

Proteins with a fold-change ≥1.5 (MCF7 vs. MCF10A), p < 0.05 (Student’s T test) are listed and grouped by molecular function. The fold change was calculated after normalization of each replicate signal to mean signal of the MCF10A positive controls as described in methods

**Table 2:** Enriched Gene Ontology Terms for MCF7 Exosome Proteins

Gene Set Name	# Genes in Gene Set (K)	# Genes in Overlap (k)	k/K	*p*-value	FDR q-value
GO_EXTRACELLULAR_SPACE	1376	44	0.032	3.80E-48	2.25E-44
GO_RECEPTOR_BINDING	1476	42	0.0285	1.61E-43	4.76E-40
GO_RESPONSE_TO_OXYGEN_CONTAINING_COMPOUND	1381	40	0.029	1.82E-41	3.58E-38
GO_POSITIVE_REGULATION_OF_RESPONSE_TO_STIMULUS	1929	42	0.0218	1.03E-38	1.53E-35
GO_RESPONSE_TO_EXTERNAL_STIMULUS	1821	41	0.0225	3.14E-38	3.72E-35
GO_REGULATION_OF_CELL_PROLIFERATION	1496	38	0.0254	5.24E-37	5.16E-34
GO_CELLULAR_RESPONSE_TO_ORGANIC_SUBSTANCE	1848	40	0.0216	1.72E-36	1.45E-33
GO_CYTOKINE_RECEPTOR_BINDING	271	24	0.0886	1.01E-35	7.49E-33
GO_IMMUNE_SYSTEM_PROCESS	1984	38	0.0192	1.85E-32	1.09E-29
GO_REGULATION_OF_CELLULAR_COMPONENT_MOVEMENT	771	29	0.0376	1.85E-32	1.09E-29

Gene Set Enrichment Analysis

To cite your use of the GSEA software, please reference Subramanian, Tamayo, et al. (2005, PNAS 102, 15545-15550) and Mootha, Lindgren, et al. (2003, Nat Genet 34, 267-273)

Among these molecules was metastasis associated protein 1 (MTA1) expressed 168-fold higher in MCF7 exosomes relative to MCF10A exosomes (*p* = 1.51E-16). MTA1 is predominantly a nuclear protein that is one of the most upregulated protein in human cancers, and associated with cancer progression [24]. In addition, MTA1 is known to regulate the hypoxia response by stabilizing HIF-1 [13]. We isolated exosomes from the normal breast cell line MCF10A, and 5 breast cancer cells lines. Exosome isolation was confirmed by detection of CD63 (a marker of exosomes) using western blot. Importantly, MTA1 was detected in exosomes derived from all 5 breast cancer cell lines including MCF7, ZR-75-1, MDA-MB-231, BT-20, and SK-BR-3 as analyzed by western blot (Fig. 1c). Cellular expression of MTA1 was analyzed by qRT-PCR (Fig. 1d)**.** These results are the first to describe MTA1 in breast cancer exosomes and the elevated levels of exosome MTA1 are consistent with reports of MTA1 expression in cancer [4–8]. We then focused on characterization of the transfer and function of exosome MTA1 in our model systems.

### Exosome MTA1 is transferred to other cells

**Fig. 2:**
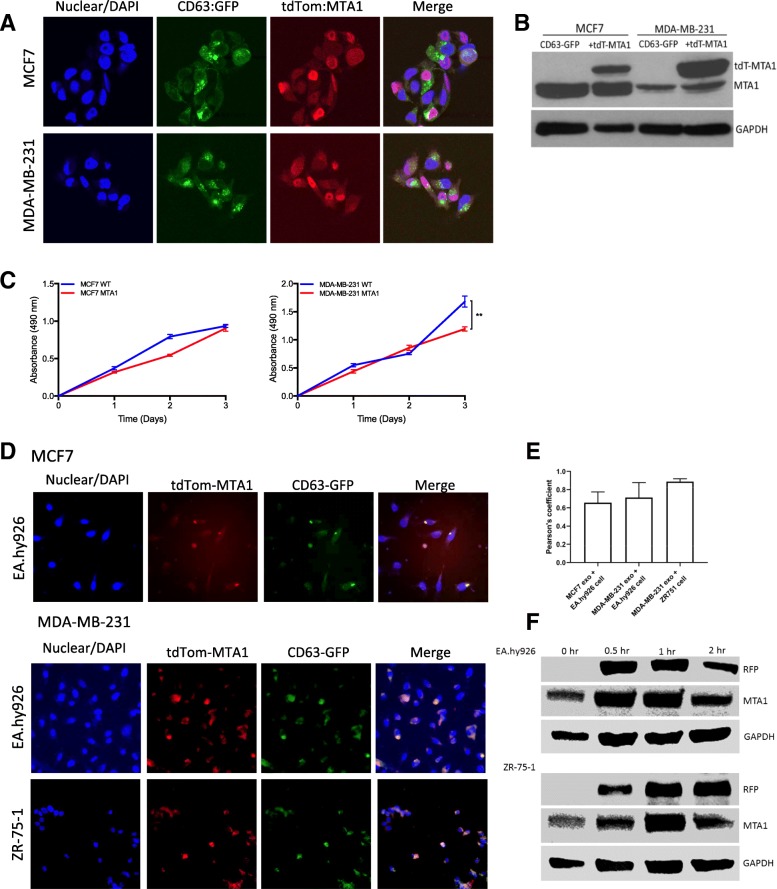
Overexpression and intercellular transfer of GFP-tagged CD63 and tdTomato-tagged MTA1 (tdTom-MTA1) in breast cancer cells. **a**. Confocal microscopy images of MCF7 and MDA-MB-231 breast cancer cells co-expressing the GFP-tagged exosome marker CD63 (CD63-GFP) and tdTomato-tagged MTA1, 40X. **b**. Western blot analysis of MTA1 expression in MCF7 and MDA-MB-231 cells expressing only CD63-GFP and tdTomato-MTA1. GAPDH is included as an equal loading control. **c**. Cell viability assay of wildtype and MTA1 overexpressing MCF7 and MDA-MB-231 cells, n = 3 **p > 0.01 Two-way ANOVA. **d**. Fluorescent microscopy imaging of CD63-GFP and tdTom-MTA1 transfer from MCF7 and MDA-MB-231 cells to endothelial (EA.hy926) and breast cancer cells (ZR-75-1) after co-culture in a transwell system for 4 days. **e**. Quantitative co-localization analysis of exosome uptake in (**d**), mean + SEM of 4 regions of interest per image. **f**. Western blot analysis of RFP (tdTom) and MTA1 uptake via exosomes isolated from MDA-MB-231 cells expressing tdTomato-MTA1. Approximately, 700–900 μg of purified exosomes were added to cultures of Ea.hy926 and ZR-75-1 cells for the indicated time. GAPDH is included as an equal loading control

We previously generated MCF7 (ER positive) and MDA-MB-231 (triple negative) breast cancer cell lines that stably express GFP-tagged CD63, a general surface marker of exosomes [17]. To visualize and track MTA1 secreted in exosomes from breast cancer cells a lentiviral vector was constructed containing tdTomato fused to MTA1 via a 3xGGGGS linker under the control of a CMV promoter (CMV-tdTom-MTA1). The CMV-tdTom-MTA1 lentiviral particles were transduced to the CD63-GFP-MCF7 and CD63-GFP-MDA-MB-231 cells. Overexpression of tdTomato-tagged MTA1 was confirmed by confocal microscopy (Fig. 2a) and western blot (Fig. 2b). The effect of tdTom-MTA1 overexpression in MCF7 and MDA-MB-231 on cell proliferation was measured. As shown in Fig. 2c, tdTom-MTA1 expression did not significantly affect the rate of proliferation of MCF7 cell lines, however the proliferation of the MDA-MB-231 cells was slightly reduced at 3 days of growth (Fig. 2c). In a trans-well co-culture system exosome-mediated transfer of MTA1 from MCF7 and MDA-MB-231 cells expressing GFP-CD63 and tdTom-MTA1 to ZR-75-1 (breast cancer cells) and EA.hy926 (vascular endothelial cells) was observed by fluorescent microscopy. As shown in Fig. 2d, co-localization of the green fluorescence (CD63-GFP) and red-fluorescence (tdTom-MTA1) is observed in both ZR-75-1 and EA.hy926 cells. The overlap of the green and red colors was quantified (Fig. 2e). Furthermore western blot confirmed the uptake of RFP (tdTom) and MTA1 by EA.hy926 and ZR-75-1 cells (Fig. 2f). These results demonstrate that MTA1 is secreted via exosomes and can be taken up by other cells.

### MTA1 CRISPR/Cas9 knockout in breast cancer cells

**Fig. 3:**
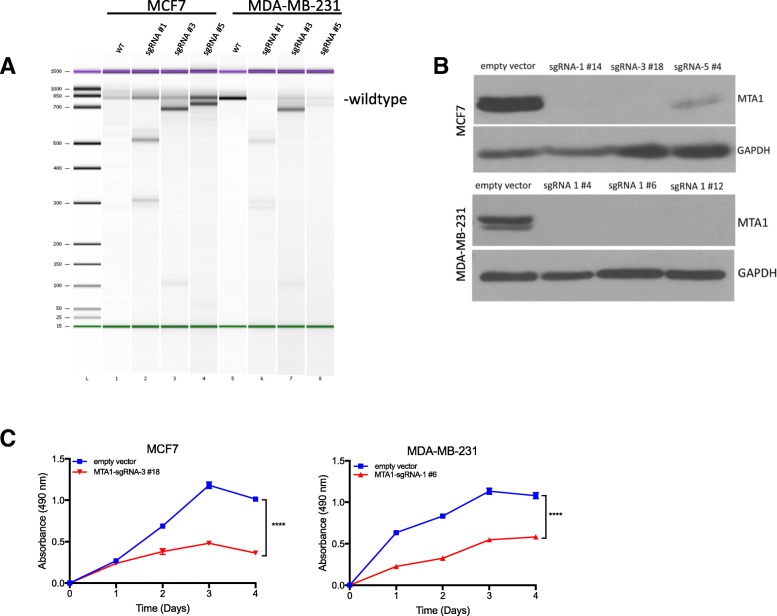
CRISPR/Cas9 deletion of MTA1 in breast cancer cells. **a**. T7 endonuclease assay PCR results. Electropherogram of the T7 endonuclease digestion of MTA1 genomic PCR products visualized on an Agilent Bioanalyzer DNA 1000 Chip. Due to location of sgRNA target site, digestion of PCR products was predicted to generate the following fragments: wildtype 800 bp, sgRNA #1: 500 and 310 bp, sgRNA#3: 700 and 100 bp, and sgRNA #5: 760 and 40 bp. **b**. Western blot of MTA1 in MCF7 and MDA-MB-231 MTA1 knockout cells. GAPDH is included as an equal loading control. **c**. Cell proliferation assay of MCF7 and MDA-MB-231 knockout cells, compared to cells expressing an empty vector, *n* = 4 ****p < 0.0001 Two-way ANOVA

In order to understand whether exosome-MTA1 contributes to breast cancer progression/metastasis, genetic knockouts of MTA1 in MCF7 and MDA-MB-231 cells were generated using a CRISPR/Cas9 expression system carrying a small guide RNA (sgRNA) targeted to MTA1. Three to five sgRNAs were designed and ligated into the lentiCRISPR v2 vector and lentiviral particles expressing Cas9 and MTA1-sgRNAs were generated and transduced to MCF7 and MDA-MB-231 cells. A T7 endonuclease assay was conducted to determine cleavage efficiency of each sgRNA. Bioanalyzer results indicated that DNA cleavage occurred with each sgRNA introduced to the cells relative to wildtype cells (Fig. 3a). After puromycin selection, MTA1-KO-MCF7 and MTA1-KO-MDA-MB-231 clones were generated and knockout was confirmed (3 clones per cell line) by western blot (Fig. 3b). Cell proliferation was significantly reduced in each MTA1 knockout breast cancer cell line relative to wildtype control cells (Fig. 3c).

### Exosome MTA1 regulates hypoxic response

**Fig. 4:**
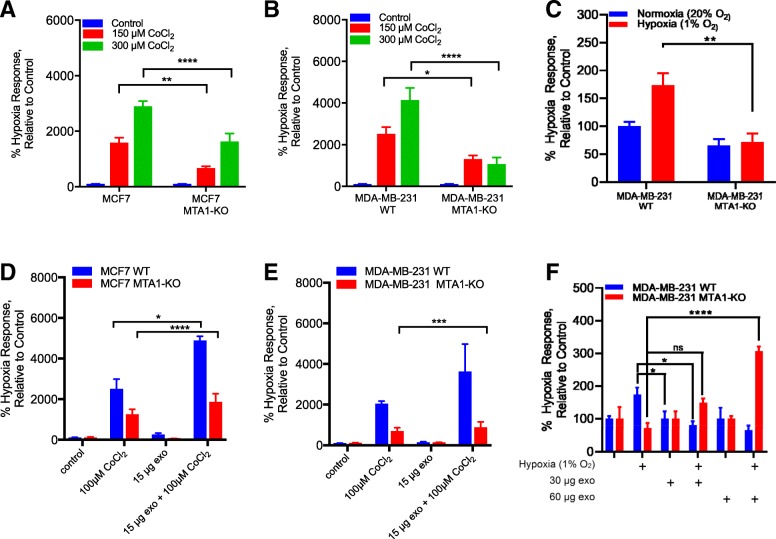
Hypoxia signaling response is attenuated in MTA1 knockout cells and restored when cells are provided with MTA1 exosomes. MCF7 (**a**) and MDA-MB-231 (**b**) wildtype and MTA1 knockout (KO) cells were transfected with the HRE reporter plasmid. Cells were treated with 150 and 300 μM CoCl_2_ for 18 h. Cells were assayed for luciferase activity. (**c**) MDA-MB-231 wildtype and MTA1 knockout were transfected with the HRE reporter plasmid and incubated in normoxic (~ 20% O2) or hypoxic (1% O2) conditions for 6 h. MCF7 (**d**) and MDA-MB-231 (**e**) wildtype and MTA1 knockout cells were pre-incubated with 15 μg of exosomes from MTA1 overexpressing MCF7 and MDA-MB-231 cells, respectively and treated with 100 μM CoCl_2_ for 18 h. (**f**) MDA-MB-231 wildtype and MTA1 knockout cells were pre-incubated with 30 or 60 μg of exosomes from MTA1 overexpressing MDA-MB-231 cells and incubated in normoxic (20% O_2_) or hypoxic (1% O_2_) conditions for 6 h. All experiments were conducted in triplicate, *p < 0.05, **p < 0.01, ***p < 0.001, ****p < 0.0001, Two-way ANOVA

As MTA1 is known to positively regulate hypoxia signaling, we sought to determine whether MTA1 overexpression or MTA1 knockout affects the hypoxic response. MCF7 wildtype, MCF7 MTA1-knockout, MDA-MB-231 wildtype and MDA-MB-231 MTA1-knockout cells were transfected with the hypoxia response element (HRE) reporter and treated with cobalt chloride for 24 h. The hypoxic response was significantly attenuated in the knockout cells relative to wildtype breast cancer cells (Fig. 4a, b). However, when purified exosomes from MTA1 overexpressing cells were pre-incubated with wildtype and MTA1 knockout cells the hypoxic response increased significantly, whereas the addition of exosomes alone (without cobalt) had no effect (Fig. 4d, e). A similar response was observed when MDA-MB-231 cells were exposed to hypoxia (1%) using a hypoxic chamber (Fig. 4c, f).

### Exosome MTA1 regulates estrogen response

**Fig. 5:**
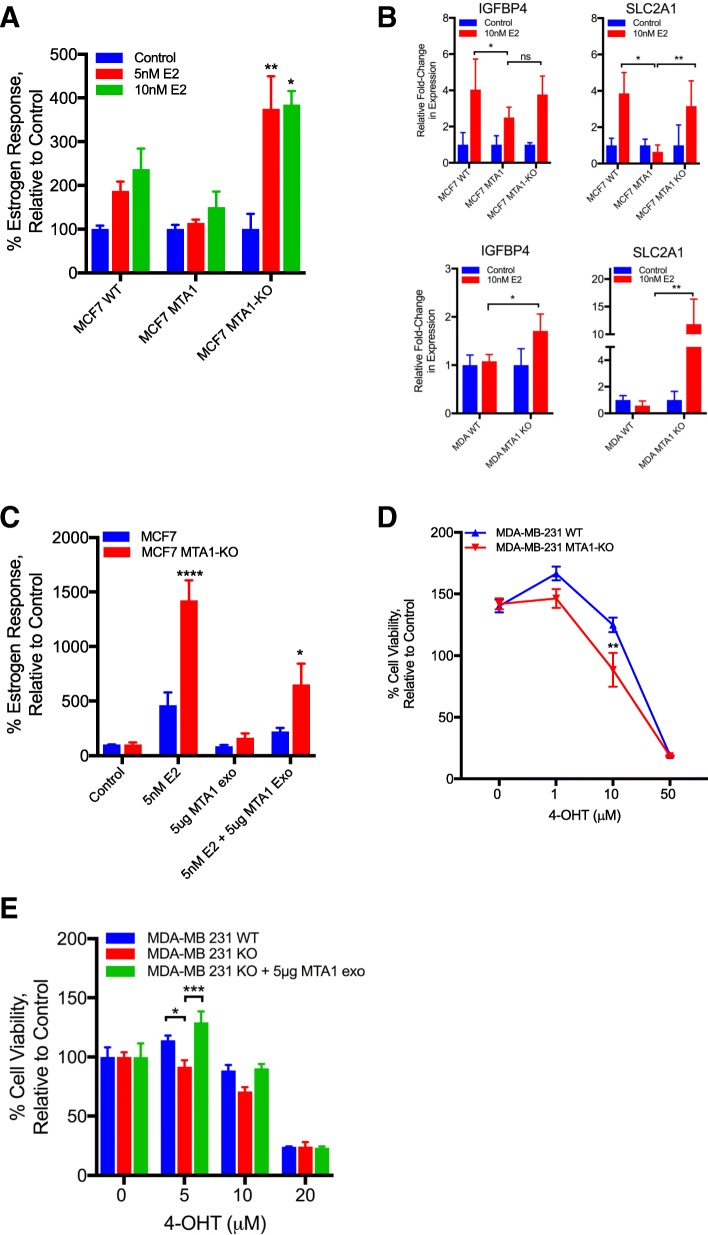
MTA1 knockout in breast cancer affects estrogen signaling and tamoxifen sensitivity that can be attenuated by the addition of MTA1 exosomes. **a** MCF7 wildtype, tdTom-MTA1, and MTA1 knockout (KO) cells were transfected with estrogen response luciferase reporter plasmid and treated with 5 or 10 nM 17β-estradiol (E2). After 24-h incubation cells were assayed for luciferase activity, *n* = 3, *p < 0.05, **p < 0.01, Two-way ANOVA. **b** Real-time PCR analysis of IGFBP4 and SLC2A1 estrogen response gene expression in wildtype, tdTom-MTA1, and MTA1-KO cells. Cells were grown in phenol-red free medium supplemented with charcoal-stripped serum for 2 days. Cells were treated with 10 nM E2 for 24 h, n = 3, *p < 0.05, **p < 0.01, Student’s t test. **c** MCF7 wildtype and MTA1-KO cells were transfected with estrogen luciferase reporter and pre-incubated with 5 μg of exosomes from tdTom-MTA1 MCF7 cells. Cells were treated with 5 nM E2 and assayed for luciferase activity after 24 h, n = 3, *p < 0.05, ****p < 0.0001, Two-way ANOVA. **d** Cell viability assay of MDA-MB-231 wildtype and MTA1 KO cells treated with 4-hydroxytamoxifen (4-OHT) at the indicated doses for 72 h, *n* = 3, **p < 0.01, Two-way ANOVA. **e** Cell viability assay of MDA-MB-231 wildtype and MTA1 KO cells were pre-incubated with 5 μg of MTA1 exosomes and then treated with 4-hydroxytamoxifen (4-OHT) at the indicated doses for 72 h, n = 3, *p < 0.05, ***p < 0.001, Two-way ANOVA

Through its interaction with histone deacetylase and the nucleosome remodeling complex, MTA1 can block the ability of estradiol to stimulate estrogen receptor-mediated transcription [10]. Therefore, we sought to determine if exosome transfer of MTA1 could also attenuate estrogen signaling. The estrogen response element luciferase reporter was expressed in MCF7 wildtype, MCF7 tdTom-MTA1, and MCF7 MTA1-knockout cells. As expected, upon stimulation with 17β-estradiol (E2), estrogen response was increased in MCF7 wildtype cells. The estrogen response was attenuated in MCF7 MTA1 overexpressing cells, while the estrogen response was significantly increased in MTA1-knockout cells as measured by luciferase reporter assay (Fig. 5a). Likewise, the expression of the estrogen signaling target genes IGFBP4 and SLC2A1 were significantly attenuated in the MTA1 overexpressing cells and in the MTA1-knockout cells expression levels were similar to the MCF7 wildtype cells (Fig. 5b**,** *upper*). Silencing of MTA1 in estrogen receptor negative cells has been shown to increase the expression of estrogen receptor α, enhance estrogen receptor signaling, and sensitize them to tamoxifen treatment [25]. Indeed, the expression of IGFBP4 and SLC2A1 were both significantly increased in MDA-MB-231 MTA1 knockout cells relative to MDA-MB-231 wildtype cells upon stimulation with E2 (Fig. 5b, *lower*). When wildtype and MTA1-knockout cells were preincubated with exosomes from MTA1 overexpressing cells, the estrogen signaling response was attenuated in both MCF7 wildtype and MTA1-knockout cells (Fig. 5c). MDA-MB-231 MTA1-knockout cells were also more sensitive to 4-hydroxytamoxifen (4-OHT) treatment than wildtype MDA-MB-231 cells (Fig. 5d). This effect could be rescued by the addition of exosomes from MTA1 overexpressing cells. (Fig. 5e). These results indicate that exosome transfer of MTA1 effects estrogen signaling response and can influence cellular response to anti-estrogen therapies.

## Discussion

The role of exosomes in cancer progression has been heavily investigated in recent years, however the predominant factors present in exosomes that may contribute to cancer development and progression via intercellular communication are not well understood. In this study, we identified many proteins in breast cancer exosomes that may contribute to breast cancer progression and specifically characterized the process and biological consequence of exosome transfer of MTA1 in breast cancer cells. We found that MTA1 is transferred through exosomes, and that exosome transfer of MTA1 alters hypoxic and estrogen signaling.

Due to its widespread overexpression in human cancers and its dual co-repressor and co-activator functions, MTA1 is considered a master regulatory molecule capable of regulating many pathways that are critical to cancer development and progression [8]. We demonstrated that MTA1 can be transferred via exosomes from breast cancer cells to *neighboring* cells and can activate or repress signaling pathways that are known to promote cancer progression. The transfer of MTA1 through breast cancer exosomes was initially revealed by a well-designed antibody array and confirmed by western blot analysis that detected MTA1 expression in exosomes derived from breast cancer cells. Definitive evidence of exosome MTA1 transfer was obtained using a co-culture model system in which a florescent-tagged MTA1 was expressed in breast cancer cells and transferred to breast cancer cells and endothelial cells. These observations indicate that even nuclear proteins can be transferred through exosomes and participate in exosome-mediated intercellular communication. Consistent with its co-activator function, exosome MTA1 was able to increase hypoxia signaling in breast cancer cells as analyzed by a reporter gene assay. Given the significance of hypoxic signaling in breast cancer, this observation strongly suggests an important role of exosome MTA1 in breast cancer progression. In line with its co-repressor function, we found that exosome MTA1 increases estrogen receptor signaling and affects tamoxifen sensitivity in triple-negative breast cancer cells, demonstrating the dual nature of this protein and the potential effect of exosome transfer of MTA1 in the tumor microenvironment. Exosome MTA1 may have to enter nucleus to alter estrogen receptor-dependent transcription. Alternatively it might suppress estrogen receptor signaling by blocking the receptor from entering nucleus. Further investigation is warranted to answer this question.

Because of its diverse roles, MTA1 is an attractive drug target, although currently no drug has been designed to specifically target MTA1. However, inhibitors that disrupt MTA1 interactions with crucial binding partners, such as histone deacetylase 1 (HDAC-1) [26, 27], have been shown to reduce metastasis and MTA1 expression in cancer cell lines and animal models [28]. The results from the present study indicate that exosome MTA1 could contribute to breast cancer progression by regulating important signaling pathways and thus may serve as a therapeutic target for the management of this malignancy. In addition, because exosomes can be isolated from various body fluids it would be of great interest to determine whether MTA1 is present in exosomes isolated from the blood of breast cancer patients or other cancer patients in general, serving as circulating biomarker for diagnosis or prognosis.

## Conclusions

This is the first study to have identified the nuclear factor MTA1 in breast cancer exosomes, demonstrated the transfer of MTA1 through exosomes, and characterized how exosome MTA1 affects cancer signaling pathways. This work suggests that exosome-mediated transfer of MTA1 is a significant driving force behind breast cancer progression and that targeting exosome MTA1 signaling or activity are potential therapeutic strategies for breast cancer management.

## Additional file

Additional file 1: Raw and normalized fluorescent intensity data. File contains the raw and normalized fluorescent intensity data from the Cancer Biomarker Antibody array. (XLSX 441 kb)

## Abbreviations

4-OHT: Hydroxytamoxifen, E2,17β-estradiol
CRISPR: Clustered regularly interspaced short palindromic repeats
ERE: Estrogen response element
HDAC-1: Histone deacetylase 1
HIF-1α: Hypoxia-inducible factor-1α
HRE: Hypoxia response element
KO: Knockout
MTA1: Metastasis-associated protein 1
NuRD: Nuclear remodeling and deacetylation
sgRNA: Small guide RNA
